# Prediction of lung cancer using gene expression and deep learning with KL divergence gene selection

**DOI:** 10.1186/s12859-022-04689-9

**Published:** 2022-05-12

**Authors:** Suli Liu, Wu Yao

**Affiliations:** College of Public Health, Zhengzhou University, Zhengzhou, 450001 China

**Keywords:** KL divergence, Gene selection, Imbalanced data, Focal loss, Deep learning, Lung cancer prediction

## Abstract

**Background:**

Lung cancer is one of the cancers with the highest mortality rate in China. With the rapid development of high-throughput sequencing technology and the research and application of deep learning methods in recent years, deep neural networks based on gene expression have become a hot research direction in lung cancer diagnosis in recent years, which provide an effective way of early diagnosis for lung cancer. Thus, building a deep neural network model is of great significance for the early diagnosis of lung cancer. However, the main challenges in mining gene expression datasets are the curse of dimensionality and imbalanced data. The existing methods proposed by some researchers can’t address the problems of high-dimensionality and imbalanced data, because of the overwhelming number of variables measured (genes) versus the small number of samples, which result in poor performance in early diagnosis for lung cancer.

**Method:**

Given the disadvantages of gene expression data sets with small datasets, high-dimensionality and imbalanced data, this paper proposes a gene selection method based on KL divergence, which selects some genes with higher KL divergence as model features. Then build a deep neural network model using Focal Loss as loss function, at the same time, we use k-fold cross validation method to verify and select the best model, we set the value of k is five in this paper.

**Result:**

The deep learning model method based on KL divergence gene selection proposed in this paper has an AUC of 0.99 on the validation set. The generalization performance of model is high.

**Conclusion:**

The deep neural network model based on KL divergence gene selection proposed in this paper is proved to be an accurate and effective method for lung cancer prediction.

## Background

Lung cancer is the malignant tumor with the highest incidence and mortality [1]. Globally, the incidence and mortality of lung cancer are increasing year by year. According to the statistics of GLOBOCAN 2020 [2], Lung cancer remained the leading cause of cancer death, lung cancer cases accounted for 11.4% of all cancer cases, and the death rate was as high as 18%. Due to the high incidence and mortality, early diagnosis of lung cancer is crucial to its cure. In the past few decades, cancer research has continued to evolve [3]. Among the various methods and research on cancer prediction, the study of gene expression level is one of the hotspots in this field. The mining of gene expression level data has promoted the early diagnosis and treatment of lung cancer. The accurate prediction of lung cancer is one of the most urgent tasks in current research [4].

Most lung cancers are diagnosed at an advanced stage, when the prognosis remains poor. Although LDCT screening has led to progress in early lung cancer detection, improvement in patient outcomes has been incremental and the accuracy is not satisfactory. With the rapid development of High-throughput sequencing, some researchers are beginning to explore the genomic evolution of premalignancy throughout the course of tumorigenesis [5]. These studies have significantly enhanced our understanding of the early molecular, cellular, and immunologic properties. Further studies are ongoing to improve early detection and develop personalized preventive therapies, which is very important for early detection and treatment of lung cancer.

With the rapid development of computer technology, machine learning methods are playing an increasingly important role in the diagnosis of lung cancer. Researchers continue to explore various lung cancer prediction algorithms, including support vector machines (SVM) [6], K-nearest neighbor (KNN) [7], Naive Bayes, but from the papers on lung cancer prediction research, these machine learning methods have various shortcomings. For example, it is difficult for SVM to find a suitable kernel function. Naive Bayes needs to know the prior probability, if the hypothetical prior distribution is not accurate, it will lead to poor prediction results.

At the same time, gene expression dataset has problems such as small dataset, high-dimensionality, unbalanced data, etc. There are tens of thousands of genes in a data set, but the number of samples is very small. Most genes have nothing to do with lung cancer, so we need exclude the irrelevant genes. If the existing machine learning methods are directly applied, the generalization performance of the model is very poor and it is difficult to converge, therefore, gene selection is needed. In the previous papers, difference analysis is usually used to select related genes. However, difference analysis requires data sets to conform to specific distributions and requires large data sets. At the same time, its calculation is more complicated, which leads to a small amount of data that cannot measure the importance of genes. There are also many researchers who try to use machine learning methods to select features, but gene expression data sets are unbalanced small data sets with high dimensionality, which leads to insufficient learning of machine learning methods and a underfitting model. The selected genes are often not the most relevant genes.

In view of the shortcomings of traditional machine learning methods and the problems of gene expression level data sets, we hope to get a new simple and convenient method for gene selection, and on this basis, we can find a new machine learning method for lung cancer prediction. In recent years some deep learning researches in the biomedical field has been successful [8], which proves deep learning [9] is an algorithm with many advantages. Compared with the traditional machine learning methods, deep learning methods do not require human experience to participate, and deep learning can well learn complex and non-linear relationships from the original data set, so this article uses deep learning methods to predict lung cancer.

In order to solve the problem of high latitude of gene expression level data and few samples, this paper tries to use KL divergence for gene selection to build a deep neural network model. In order to solve the problem of imbalanced data, focal loss [10] is used as loss function. We use the output of the deep neural network model as the final prediction result. The final results show that the deep neural network model based on KL divergence gene selection proposed in this paper has obtained a relatively high AUC on the LUAD data set of TCGA [11] and ICGC, which can accurately diagnose lung cancer.

## Methods

**Fig. 1:**
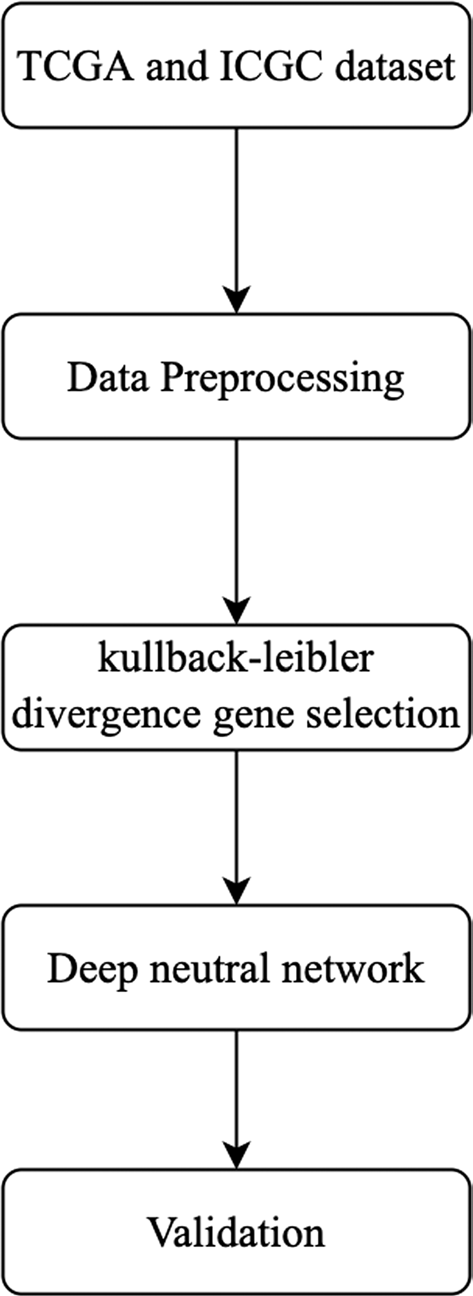
Flowchart of the proposed Kullback–Leibler divergence gene selection-based deep learning method. The process of the deep neural network lung cancer prediction model based on KL divergence gene selection proposed in this paper

The process of the deep neural network lung cancer prediction model based on KL divergence gene selection proposed in this paper is shown in Fig. 1. Firstly, we use KL divergence to select the related genes to lung cancer as the input of the deep neural network lung cancer prediction model. Secondly, we build a deep neural network which uses focal loss as the loss function and use the training set to train the model. Finally, we use the validation set to verify the generalization performance of the lung cancer prediction model and select a prediction model with the best parameters.

### Data collection and preprocessing

The data we used were extracted from TCGA portal (https://tcga‐data.nci.nih.gov/tcga/) and ICGC portal (https://dcc.icgc.org/). The TCGA dataset and ICGC dataset used in this paper is the RNA-seq gene expression data of lung adenocarcinoma (LUAD) samples from the TCGA dataset. The TCGA dataset contains 533 lung cancer samples and 59 normal samples. The ICGC dataset contains 488 lung cancer samples and 55 normal samples.

This paper uses python to process the data into a training format that TensorFlow and sklearn can recognize.

### KL divergence gene selection

There are more than 60,000 genes in the RNA-seq data in the TCGA and ICGC database, and more than 20,000 genes with protein translation. When using too much genetic data to train a lung cancer prediction model, it is easy to overfit. In clinical practice, the number of available cancer samples is very small compared to the number of gene features, which leads to model overfitting and decreased prediction accuracy. Feature selection is a good way to deal with these problems [12]. By reducing the entire feature space to a subset of selected features, over-fitting of the prediction model can be avoided, thereby reducing the problems caused by small sample sizes and high-latitude data. We mentioned above that the existing differential analysis gene selection methods and machine learning-based gene selection methods have some shortcomings [13]. For example, differential analysis gene selection methods have requirements for data distribution, and gene selection methods based on machine learning require a lot of data, otherwise it is easy to overfit. Taking into account the shortcomings of the above methods, this paper proposes a gene selection method based on KL divergence.

KL divergence [14] (Formula (1)) is an asymmetry measure of the difference between two probability distributions over the same variable *x*(P and Q represent two data distributions). In practice, P represents the true distribution of the data, and Q represents the theoretical distribution of the data or the approximate distribution of P.

1 $$\begin{array}{*{20}c} {{\text{D}}_{{{\text{kl}}}} = - \mathop \sum \limits_{i = 1}^{i = n} P\left( i \right)*ln\frac{Q\left( i \right)}{{P\left( i \right)}} \ge 0} \\ \end{array}$$

The KL divergence is always greater than or equal to zero. When the two data distributions are the same, the value of the KL divergence is 0. The greater the difference between the two distributions, the greater the value of the KL divergence.

For gene expression data, we can easily get the data distribution of each gene in the disease group and the control group. We can easily get the data distribution using a small sample data set, and then use KL divergence to measure the difference between the two distributions. If the two distributions are consistent, it means that the gene has nothing to do with the disease. If the two distributions are quite different, it means that the gene is related to the disease.

KL divergence has the advantage of simple calculation. We can easily calculate the difference between the two distributions using small data set, which is suitable for small dataset such as gene expression dataset.

### Building deep natural network model

This paper uses a deep neural network model to predict lung cancer. Deep neural network is inspired by the working principle of the brain and has been widely used in many fields. A deep neural network generates output based on input variables. Given a set of features and a target, it can learn to generate nonlinear function approximations value. Between the input and output, there are one or more nonlinear layers, called hidden layers. The deep neural network has multiple nonlinear hidden layers, which enable the deep neural network to learn complex nonlinear function relationships from high-dimensional raw data without the guidance of artificial rules [9].

**Fig. 2:**
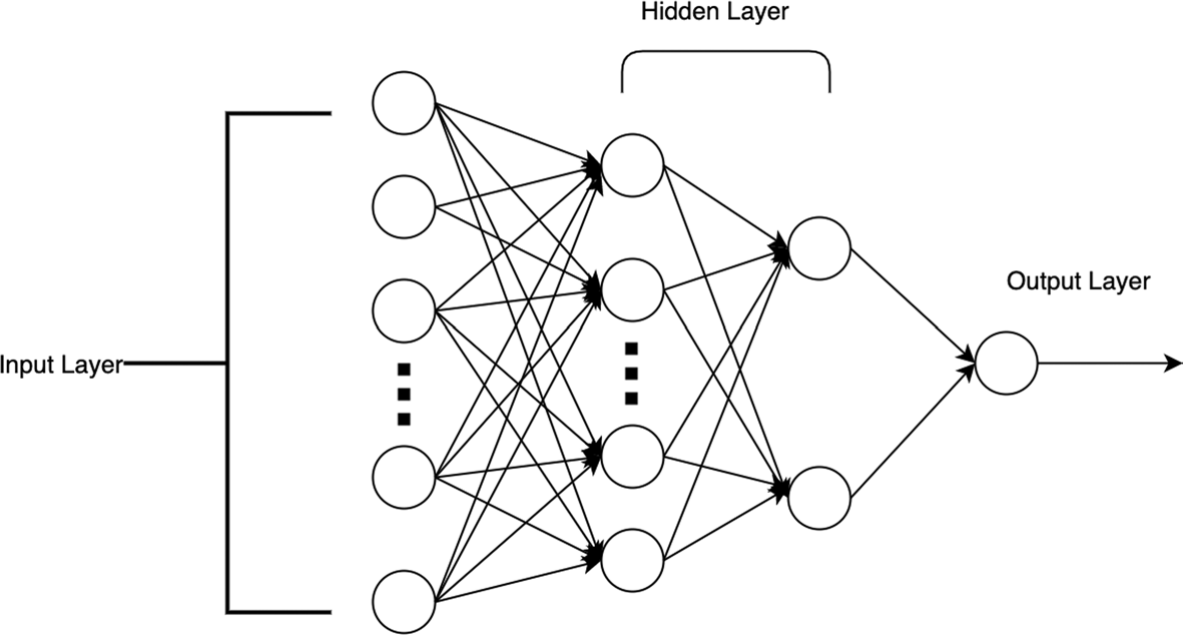
The illustration of the neural network structure. The structure of a deep neural network, which contains two hidden layers and the output layer is a sigmoid function

Figure 2 shows the deep neural network model constructed in this paper. The leftmost layer is the input layer, the rightmost layer is the output layer, and the middle layer is a hidden layer composed of hidden neurons. Then we set the loss function that meets your needs. Gradually reducing the loss value during the training process achieves the purpose of model convergence. The specific formulas of the model inference process are shown in Formula (2) to Formula (6).

2 $$\begin{array}{*{20}c} {hidden\_layer\_1 = relu\left( {input*W_{1} } \right)} \\ \end{array}$$

3 $$\begin{array}{*{20}c} {hidden\_layer\_2 = relu\left( {hidden\_layer\_1*W_{2} } \right)} \\ \end{array}$$

4 $$\begin{array}{*{20}c} {\hat{y} = sigmod\left( {hidden\_layer\_2} \right)} \\ \end{array}$$

5 $$\begin{array}{*{20}c} {relu\left( x \right) = max\left( {x,0} \right)} \\ \end{array}$$

6 $$\begin{array}{*{20}c} {sigmod\left( x \right) = \frac{1}{{1 + e^{ - x} }}} \\ \end{array}$$

Formula (2) and Formula (3) are the calculations of the hidden layer, where *input* is the input information. *W*_1_ is the parameter of the first layer, *relu*(*x*) is a non-linear function, which is defined as Formula (5). Formula (4) is the predicted output value of the model, using the *sigmod* function as the activation function[15]. The *sigmod* function is defined as shown in Formula (6), and its output value range is 0–1, which conforms to the meaning of probability, so the output value is the model’s prediction probability.

The binary classification model generally uses cross-entropy as the loss function, as shown in Formula (7), where y represents whether the sample is diseased, the disease is 1, and the non-diseased is 0, and $\hat{y}$ represents the estimated sample's disease probability, we can see that the smaller the model classification error, the smaller the value of the loss function Formula (7). The cross-entropy loss function can achieve better results on a balanced data set, however, the gene expression data set is unbalanced, it is easy to distinguish the number of samples is relatively too large, and ultimately dominates the total loss, leading to the prediction result tends to be a large number of parties.

7 $$\begin{array}{*{20}c} {loss = - y*log\left( {\hat{y}} \right) - \left( {1 - y} \right)*log\left( {1 - \hat{y}} \right)} \\ \end{array}$$

In order to solve the problem of imbalanced data, we use focal loss function. Its formula is as shown in Formula (8). Focal loss considers that samples that are easily distinguishable by the model (samples with high confidence) have a very small improvement performance on the model. The model should pay attention to samples that are not easy to distinguish, at the same time adjust the ratio of positive and negative samples, which is reflected in the parameter α can adjust the ratio of positive and negative samples, and parameter γ can adjust the weight of samples that are easy to distinguish and improve the weight of the samples that are not easy to distinguish.

8 $$\begin{array}{*{20}c} {loss = - y*\alpha *\left( {1 - \hat{y}} \right)^{{\upgamma }} *log\left( {\hat{y}} \right) - \left( {1 - y} \right)*\widehat{{y^{{\upgamma }} }}*log\left( {1 - \hat{y}} \right)} \\ \end{array}$$

We use the gradient descent method to adjust W1 and W2. Taking into account the shortcomings of existing methods and the characteristics of gene expression data sets, this paper uses TensorFlow to establish a three layers deep neural network and uses Adam as the gradient descent optimizer [16]. In order to solve the problem of imbalanced data, this paper uses focal loss as a loss function, and multiple rounds of training were performed on the training data set.

## Results

### Dataset

We evaluated the proposed method using k-fold cross validation method on the LUAD RNA-seq dataset, which comes from the TCGA dataset and ICGC dataset. These data sets include all stages of lung cancer, collected from patients of different clinical conditions and different ages and genders. The specific information of the data set is shown in Table 1. In the data preprocessing stage, we use ENSEMBL gene annotation files to select genes whose biotype is protein coding. we use seq-count as gene expression level data for gene selection and model training.

**Table 1:** Dataset information

Data set	Genes	Tumor samples	Normal samples	Total samples
TCGA-LUAD	19,565	533	59	592
ICGC-LUAD	19,565	488	55	543

### Gene selection

This article analyzes the gene expression distribution of each gene in normal group and lung cancer group and calculates the KL divergence of two distributions. We believe that genes with larger KL divergence are genes that are related to lung cancer.

After analysis and comparison, setting the KL divergence threshold to 3 can select lung cancer-related genes, the number of selected genes is small. In this paper, a total of 194 genes were selected.

We use Accuracy, Recall, Precision to evaluate the performance of gene selection models and non-gene selection models. These indicators all rely on the confusion matrix [11] for calculation. The definition of confusion matrix is shown in Table 2.

**Table 2:** Confusion matrix

Confusion matrix	Predicted	
	Positive	Negative
*Actual*		
Positive	True positive	False negative
Negative	False positive	True negative

The accuracy rate is defined as the proportion of the predicted correct samples in the total samples, and the calculation formula is shown in Formula (9).

9 $$\begin{array}{*{20}c} {Accuracy = \frac{TP + TN}{{TP + FP + TN + FN}}} \\ \end{array}$$

The recall rate is defined as the proportion of all samples whose true values are positive that are predicted to be correct. The calculation formula is shown in Formula (10).

10 $$\begin{array}{*{20}c} {Recall = \frac{TP}{{TP + FN}}} \\ \end{array}$$

The precision is defined as the proportion of samples whose true values are positive among all samples whose predicted values are positive. The calculation formula is shown in Formula (11).

11 $$\begin{array}{*{20}c} {Precision = \frac{TP}{{TP + FP}}} \\ \end{array}$$

We use the deep neural network model proposed in this article to train and evaluate all gene data and selected gene data respectively. The results are shown in Table 3. We can see that the accuracy of the model is further improved after feature selection. At the same time, the tradeoff between recall rate and accuracy rate is better, the classification performance of feature selection data is more stable, and the calculation time is shorter. Therefore, we will use the selected data as the input for the subsequent process, so that we can get more accurate lung cancer prediction, less model training time, and smaller model size.

**Table 3:** The precision, recall, accuracy and train time of the entire data and selected data analyzed by proposed method

Dataset	Feature type	Precision (%)	Recall (%)	Accuracy (%)	Time of training (min)
TCGA-LUAD	Entire data	98.16	100	98.07	120
	Selected data	99.87	100	99.93	10
ICGC-LUAD	Entire data	98.34	100	98.13	120
	Selected data	99.96	100	99.96	10

### Deep neural network prediction model based on gene selection

We built a deep neural network model with two hidden layers, using focal loss as the loss function, taking the genes selected in the previous section as the model input, and then using k-fold cross validation method to evaluate the performance of the model, we set the value of k is five. After feature selection, the dimensionality of the data set is reduced, much lower than the sample size, which makes the application of deep neural network models possible.

**Fig. 3:**
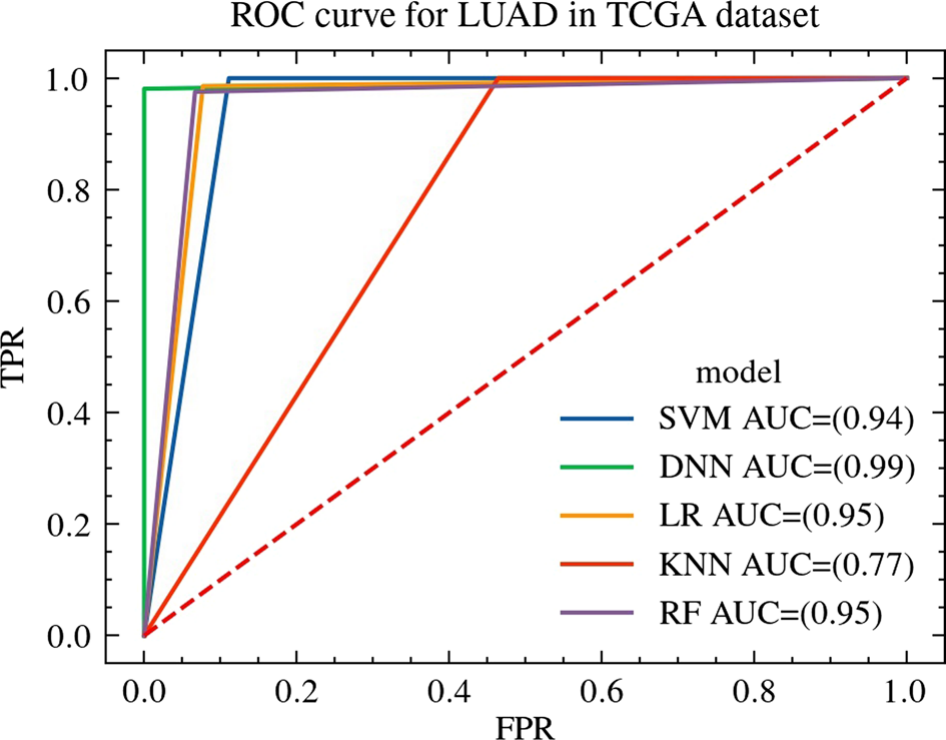
ROC curve for LUAD in TCGA dataset. ROC curve for LUAD in TCGA dataset, where the abscissa is false positive rate, the ordinate is true positive rate, and the area below the ROC curve is AUC

**Fig. 4:**
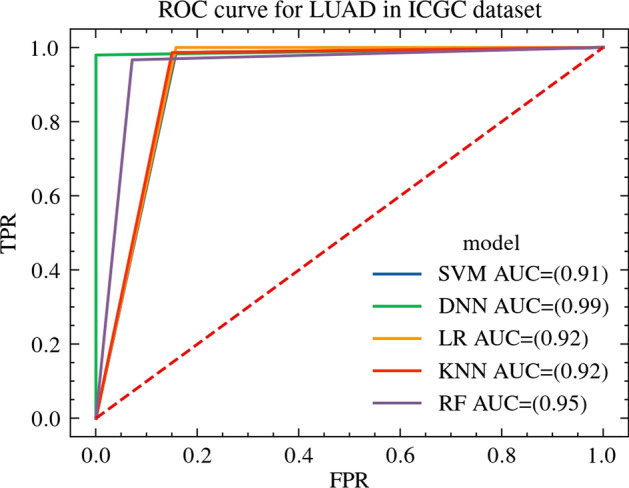
ROC curve for LUAD in ICGC dataset. ROC curve for LUAD in ICGC dataset, where the abscissa is false positive rate, the ordinate is true positive rate, and the area below the ROC curve is AUC

Compared with the SVM[17], LR, KNN and RF methods, the AUC of the deep neural network model based on feature selection proposed in this paper on the LUAD and ICGC dataset is higher. AUC is the area under the ROC curve, indicating the performance of the binary classification model, indicating the probability that the model will rank positive samples in front of negative samples, which can well reflect the accuracy and recall rate of the model. In the data set of this paper, the ROC curves of SVM, LR, KNN, RF and the model in this paper are shown in Figs. 3 and 4. The Fig. 3 show the ROC of all models trained in TCGA dataset, The Fig. 4 show the ROC of all models trained in ICGC dataset.

The AUC of the SVM, LR, KNN, RF model and the model proposed by this paper on TCGA and ICGC dataset are shown in Table 4. From the table, we can get that due to the non-linear fitting ability and automatic feature intersection ability of the deep learning model, the AUC of the model proposed by this paper is significantly higher than the other models, and the prediction accuracy is very high.

**Table 4:** The AUC for LUAD data

Classification algorithm	AUC on ICGC data	AUC on TCGA data
SVM	0.91	0.94
LR	0.92	0.95
KNN	0.92	0.77
RF	0.95	0.95
Proposed method	0.99	0.99

## Discussion

In this paper, we found that the deep neural network model based on feature selection proposed in this paper has achieved better results than normal classification models in lung cancer prediction. Given the high incidence and mortality of lung cancer, early and accurate detection is very important. Therefore, computer artificial intelligence technology is of great help to improve the accuracy of lung cancer prediction.

In this paper, we compare the deep learning model proposed in this paper with the SVM model. The SVM model has been widely used in disease prediction. The SVM can achieve better results on a balanced big data set, but on the gene expression data set, the generalization performance of SVM is poor. This is probably because the feature dimension is too high and the SVM cannot be fully learned, resulting in low prediction accuracy. Therefore, feature selection for high-dimensional data can reduce the cost of model learning, which makes it easier for the model to converge, so we can obtain better prediction accuracy.

In addition, we observed that our deep learning-based model achieved higher accuracy and AUC scores than SVM. The result may be attributed to the inability of SVM to perform automatic feature crossover. The deep learning method we proposed can automatically learn the direct interaction and non-linear relationship of features and perform the best fit. Therefore, the accuracy of lung cancer prediction is higher. Our research results also confirm that deep learning has the ability to fit complex relationships, especially non-linear relationships, and does not require manual intervention. We believe that deep learning will become more and more important in the field of disease diagnosis and has a broad space for development.

Finally, we need to point out that deep learning-based models require high computational costs. In order to overcome this problem, we use feature selection techniques in the data preprocessing stage, which greatly reduces model training and running time and makes the model easier to converge. With the rapid increase in the amount of gene expression data and the diversity of features, feature selection is a very important and necessary means. In general, in the discovery and research of important genes, feature selection is more and more worthy of attention.

## Conclusions

Aiming at the shortcomings of existing lung cancer prediction methods, this paper proposes a lung cancer prediction model based on KL divergence gene selection using deep neural network, which can solve the problem of high dimensionality, few samples, and model unfitting on gene expression data. Compared with traditional algorithms, it has the advantages of fast training and high accuracy, and it performs better on the verification data set.

## Data Availability

The TCGA data analyzed in the current study is level-3 data, which are publicly available in the National Cancer Institute Genomic Data Commons Data Portal, https://portal.gdc.cancer.gov/. The data does not need any administrative permissions to access. The ICGC data analyzed in the current study is publicly available in international Cancer Genome Consortium Data Portal, https://dcc.icgc.org/. The data does not need any administrative permissions to access. The full and transformed gene expression level data, and our code for running these analyses are available via GitHub at https://github.com/liusulizzu/cancer_prediction_tcga.
